# FOXO4 expression is related to stem cell-like properties and resistance to treatment in diffuse large B-cell lymphoma

**DOI:** 10.18632/oncotarget.13690

**Published:** 2016-11-29

**Authors:** Kyung Ju Ryu, Chaehwa Park, Mineui Hong, Young Hyeh Ko, Won Seog Kim, Seok Jin Kim

**Affiliations:** ^1^ Department of Health Sciences and Technology, Samsung Advanced Institute for Health Sciences and Technology, Sungkyunkwan University, Seoul, Korea; ^2^ Samsung Biomedical Research Institute, Samsung Medical Center, Seoul, Korea; ^3^ Department of Pathology, Kangnam Sacred Heart Hospital, Hallym University Medical Center, Seoul, Korea; ^4^ Department of Pathology, Samsung Medical Center, Sungkyunkwan University School of Medicine, Seoul, Korea; ^5^ Division of Hematology and Oncology, Department of Medicine, Samsung Medical Center, Sungkyunkwan University School of Medicine, Seoul, Korea

**Keywords:** stem cell, FOXO4, resistance, B-cell, lymphoma

## Abstract

Cancer stem cells are proposed to be responsible for resistance to chemotherapeutic agents, including doxorubicin. As phenylbutyrate enhances cancer stem cell properties, we analyzed surviving lymphoma cells after treatment with doxorubicin and phenylbutyrate. Human B-cell lymphoma cell lines, including Toledo, BJAB, Daudi, and Raji were incubated with IC_90_ concentrations of doxorubicin (300 nM) or phenylbutyrate (8 mM). After 48 h, live cells were sorted and analyzed for their resistance to treatment by examining gene expression profiles using cDNA microarray and biological characteristics. A small fraction of lymphoma cells that survived after drug application showed higher expression of stem cell markers (*NANOG*, *and* *SOX2*) and superior ability of self-renewal and sphere formation, compared to untreated control cells (*P* < 0.05). Gene expression analysis disclosed elevated expression of 41 genes, including *FOXO4*, in the four lymphoma cell lines that survived drug treatment. Overexpression of *FOXO4* was evident in lymphoma cells surviving after phenylbutyrate treatment and refractory patient-derived lymphoma cells. Induction of *FOXO4* expression promoted self-renewal whereas its knockdown led to diminished expression of stem cell markers and colony-forming ability of lymphoma cells. Immunohistochemical staining for FOXO4 in tumor tissue of diffuse large B-cell lymphoma revealed nuclear localization and significant association with poor prognosis. In conclusion, lymphoma cells resistant to treatment exhibit stem cell-like properties and enhanced FOXO4 expression. The presence of FOXO4-expressing cells in tumor tissue and their association with poor survival supports a role of FOXO4 in promoting stem cell properties resulting in poor outcomes.

## INTRODUCTION

Diffuse large B-cell lymphoma (DLBCL) is the most common subtype of non-Hodgkin lymphoma accounting for ~30–40% of lymphomas in Western and Asian countries [1]. DLBCL is a curable disorder by systemic chemotherapy because lymphoma cells are sensitive to chemotherapy. However, a substantial number of patients still fail to be cured and these treatment failures are mainly associated with drug resistance. Lymphoma cells could acquire drug resistance during they were repeatedly exposed to chemotherapeutic agents. However, a subset of cells with a peculiar characteristics leading to primary refractoriness to chemotherapy might exist at diagnosis even though it has never been treated with chemotherapeutic agents. The concept of cancer stem cells (CSC) has been proposed to explain the existence of a cell population responsible for tumorigenesis as well as primary refractoriness to treatment in patients [2–4]. The CSC hypothesis postulates that this specific cell population can self-renew and produce daughter cells, continuously leading to treatment failure [5]. Consistently, the association of this cell population with poor prognosis has been suggested in several cancer types, including breast, brain and colon cancer, following the first report on the presence of CSCs in acute myeloid leukemia [6–10]. Thus, accumulating data support the involvement of CSCs in treatment resistance resulting in tumor relapse and metastasis [11, 12]. At present, limited data on CSCs in DLBCL are available due to a lack of definitive markers, although a few potential markers have been identified in other B-cell lymphomas [13, 14].

In the current study, we developed an *in vitro* model mimicking a cell population that is primarily refractory to treatment by isolating a cell subset that survived after treatment with the drug at IC_90_ concentrations (required for 90% inhibition of tumor cell growth). Given that surviving cells after long-term exposure to low-dose drug may represent those cells with acquired rather than intrinsic resistance, we treated cells with high concentrations of drug for a short duration of time. Doxorubicin and phenylbutyrate were used for drug treatment, since doxorubicin is the main chemotherapeutic agent in various regimens for DLBCL and phenylbutyrate is a histone deacetylase inhibitor reported to induce stemness in human induced pluripotent stem cells [15]. Gene expression profiles of the surviving cell population revealed consistent overexpression of forkhead box O 4 (*FOXO4*), compared with the control group. Although FOXO transcription factors are considered tumor suppressors, contributory roles to cancer progression and maintenance of CSCs in acute and chronic myeloid leukemia have been recently described [16–18]. Accordingly, we examined the potential function of *FOXO4* in B-cell lymphoma cell populations showing stem cell-like properties, and demonstrated its prognostic value in DLBCL patients.

## RESULTS

### Generation of B-cell lymphoma cells surviving drug treatment

**Figure 1:**
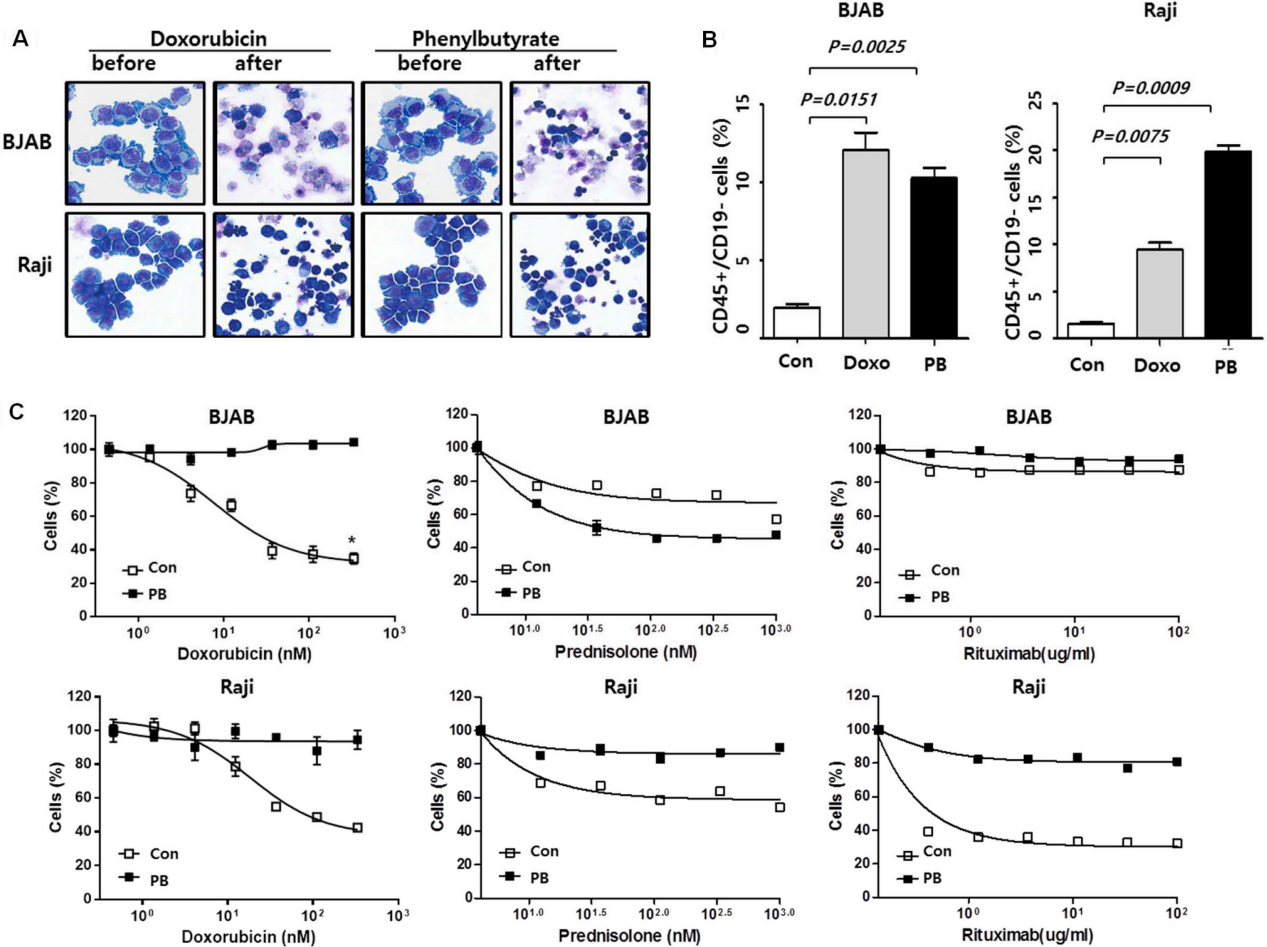
Generation of B-cell lymphoma cells surviving drug treatment (**A**) Morphology of BJAB and Raji cells after 48 h incubation with doxorubicin (300 nM) or phenylbutyrate (8 mM): Original magnification, x 400; May–Grünwald–Giemsa staining. (**B**) Flow cytometry analysis of the CD45+/CD19− cell population and comparison of CD45+/CD19− cell fraction among control cells (con), doxorubicin (Doxo) and phenylbutyrate (PB)-treated surviving cells. (**C**) Dose-response curves shows higher viability of phenylbutyrate (PB)-treated surviving BJAB and Raji cells than control cells (con) when cells are seeded at a density of 5 × 10^4^ cells per well in 24-well plates, treated with the indicated doses of doxorubicin, prednisolone and rituximab. Data represents means ± SEM of three independent experiments.

Seven lymphoma cell lines (BJAB, Raji, Daudi, Toledo, OCI-Ly10, RIVA, and U2932) were treated with the IC_90_ dose of doxorubicin (300 nM) or phenylbutyrate (8 mM) for 48 h. The majority of cells died after treatment with a few surviving cells, and the proportions of viable cells are specified in Supplementary Table S1. The morphology of lymphoma cells surviving after 48 h incubation with doxorubicin (300 nM) or phenylbutyrate (8 mM) was different from control cells, and their immunophenotype was also different (Figure 1A, 1B). The comparison of immunophenotype using B-cell marker, CD19 showed both groups, surviving cells after treatment with doxorubicin and phenylbutyrate had significantly higher number of CD19-negative cells than control groups. Thus, the proportion of CD45+/CD19− cells which was previously reported as CSC of B-cell lymphoma was significantly higher in surviving cells than control cells (Figure 1B) [13, 14]. Given the nature of drug resistance of surviving cells after IC_90_ dose of phenylbutyrate (PB cells), drug sensitivity was analyzed. Compared to control cells, BJAB-PB and Raji-PB cells showed higher viability when they were exposed to various concentrations of doxorubicin, prednisolone and rituximab (Figure 1C). Especially, the median inhibitory concentrations (IC50) of doxorubicin were 28.04 and 39.33 nM for BJAB and Raji control cells whereas those for BJAB-PB and Raji-PB cells were over 300 nM (*P* < 0.05). Thus, phenylbutyrate-treated surviving cells showed resistance to other anti-lymphoma agents.

### Stem cell-like properties of B-cell lymphoma cells surviving drug treatment

**Figure 2:**
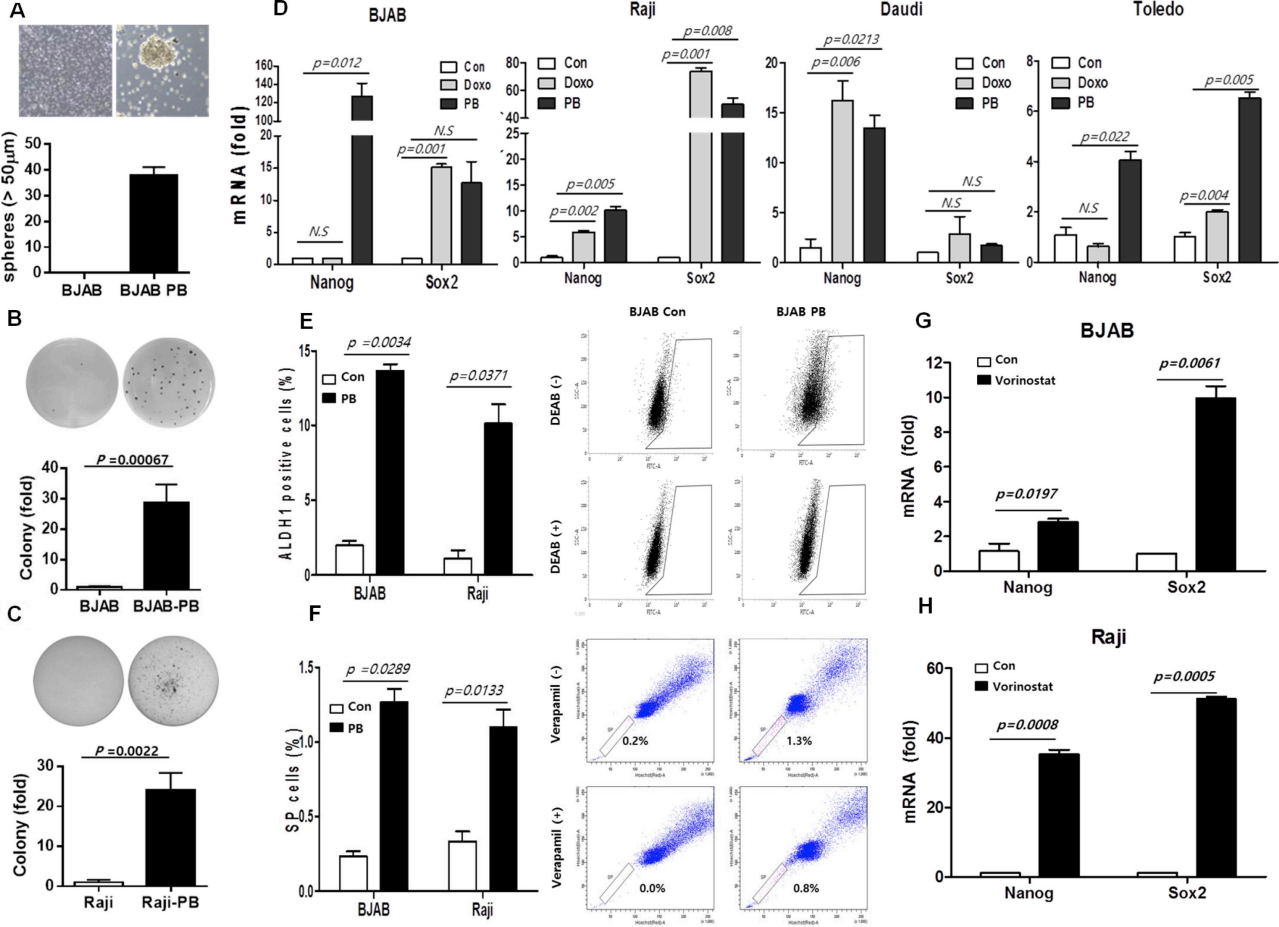
Stem cell-like properties of B-cell lymphoma cells surviving drug treatment (**A**) Phenylbutyrate-treated surviving cell (PB) shows tumor sphere formation in BJAB-PB cells compared to BJAB cells. (**B, C**) Colony formation is increased in BJAB-PB and Raji-PB cells compared to control cells. (**D**) Stem cell marker mRNA levels (Nanog and Sox-2) are increased in lymphoma cells surviving treatment with doxorubicin (Doxo) or phenylbutyrate (PB) compared to control cells (Con) in BJAB and Raji. Data represent means ± SEM of three independent experiments. (**E**) BJAB-PB and Raji-PB cells show significantly higher proportion of aldehyde dehydrogenase (ALDH)-positive cells than control cells (Con). (**F**) BJAB-PB and Raji-PB cells show more increased fraction of side-population than control cells. (**G, H**) Another histone deacetylase inhibitor, vorinostat-treated surviving cells show higher mRNA level of stem cell markers (Nanog and Sox-2) than control cells.

Because CSC could be related to drug resistance and tumor sphere formation is a surrogate marker of self-renewal of cancer stem cells, we sorted live cells via flow cytometry and plated them in stem cell-selective conditions to observe formation of spheres. As a result, cells surviving after phenylbutyrate treatment generated significantly higher number of tumor spheres compared to control cells (Figure 2A). As phenylbutyrate is known to induce stem cell-like properties in mature tumor cells [15], we further evaluated stem cell-like properties in phenylbutyrate-treated surviving cells. In the soft agar colony formation assays, PB cells showed greater colony formation than control cells (Figure 2B, 2C). In accordance with these findings, the expression of stem cell markers (NANOG and SOX2) was significantly higher in B-cell lymphoma cells survived after phenylbutyrate and doxorubicin treatment than control cells (Figure 2D). This increased expression of stem cell markers was also observed in other B-cell lymphoma cell lines survived after phenylbutyrate and doxorubicin treatment (OCI-Ly10, Riva, and U2932, Supplementary Figure S1). Moreover, BJAB-PB and Raji-PB cells exhibited slower proliferation rates than control cells (Supplementary Figure S2). In consistent with these stem cell-like properties, the BJAB-PB and Raji-PB cells showed significantly higher proportion of aldehyde dehydrogenase (ALDH)-positive cells than control cells (Figure 2E) because ALDH is known as a marker for CSC [19]. The BJAB-PB and Raji-PB cells also showed the enrichment of side-population compared to control cells (Figure 2F), thus, these cell populations represented CSC like previous reports [20, 21]. When we applied another histone deacetylase inhibitor, vorinostat on B-cell lymphoma cells with the same manner, the similar effect of vorinostat on the stemness of lymphoma cells was observed in BJAB and Raji cells (Figure 2G, 2H).

### Analysis of gene expression profiles and *FOXO4* overexpression

**Figure 3:**
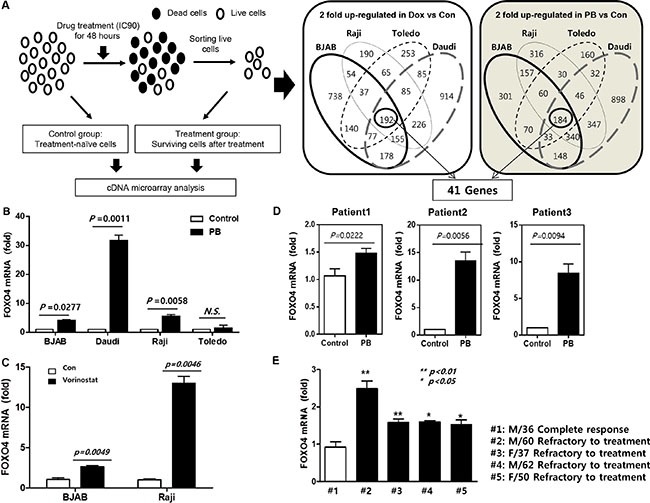
Comparison of gene expression profiles and FOXO4 overexpression (**A**) Study scheme of gene expression analyses using doxorubicin (Dox) and phenylbutyrate (PB)-treated surviving cells: Four different cell lines (BJAB, Raji, Toledo, and Daudi) are treated with doxorubicin (300 nM) or phenylbutyrate (8 mM) for 48 h, and cDNA microarray analysis is done to identify differentially expressed target genes between treatment-surviving cells and parental control cells. (**B**) *FOXO4* mRNA level is significantly higher in phenylbutyrate-treated surviving (PB) cells of BJAB, Raji and Daudi than control cells. (**C**) Vorinostat-treated surviving cells show higher mRNA level of *FOXO4* than control (Con) cells of BJAB and Raji. (**D**) Primary lymphoma cells from three patients with refractory diffuse large B-cell lymphoma (DLBCL) show increased expression of *FOXO4* in phenylbutyrate-treated surviving cells (PB) compared to the corresponding control cells. (**E**) The *FOXO4* mRNA level is significantly higher in primary cells from four patients with refractory DLBCL than that of a patient with DLBCL who achieved complete response. Data represent means ± SD from three independent experiments.

The cDNA microarray analysis was performed to identify the differentially expressed target genes between treatment-surviving and corresponding control cells (Figure 3A). Comparison of the gene expression profiles in four lymphoma cell lines (BJAB, Raji, Toledo, and Daudi) revealed that 41 genes are commonly overexpressed in cells surviving treatment with doxorubicin or phenylbutyrate, compared with untreated control cells (Supplementary Table S2). Among 41 genes, *FOXO4* was one of the candidate genes identified because FOXO transcription factors, which are directly inhibited by Akt-dependent phosphorylation, have been implicated in stem cell maintenance of several cell lineages [22]. Increased expression of *FOXO4* in phenylbutyrate-treated surviving cells was confirmed in three B-cell lymphoma cell lines (BJAB, Raji and Daudi) and this increased expression of *FOXO4* was also found in vorinostat-treated surviving cells (Figure 3B, 3C). *FOXO*4 expression was also significantly enhanced in surviving cells from other DLBCL cell lines (OCI-Ly10, Riva, and U2932) (Supplementary Figure S3). The increased expression of *FOXO4* was found in B-cell lymphoma patient-derived cells. Thus, when three patient-derived primary cells were treated with phenylbutyrate in the same method as cell lines, phenylbutyrate-treated surviving cells showed significant higher expression of *FOXO4* than control cells (Figure 3D). Moreover, primary cells obtained from malignant ascites or pleural effusion of refractory B-cell lymphoma patients showed significantly more increased expression of *FOXO4* than that of a patient who completely responded to 1st line treatment (Figure 3E).

### Effect of FOXO4 on stem cell properties of treatment-surviving cells

**Figure 4:**
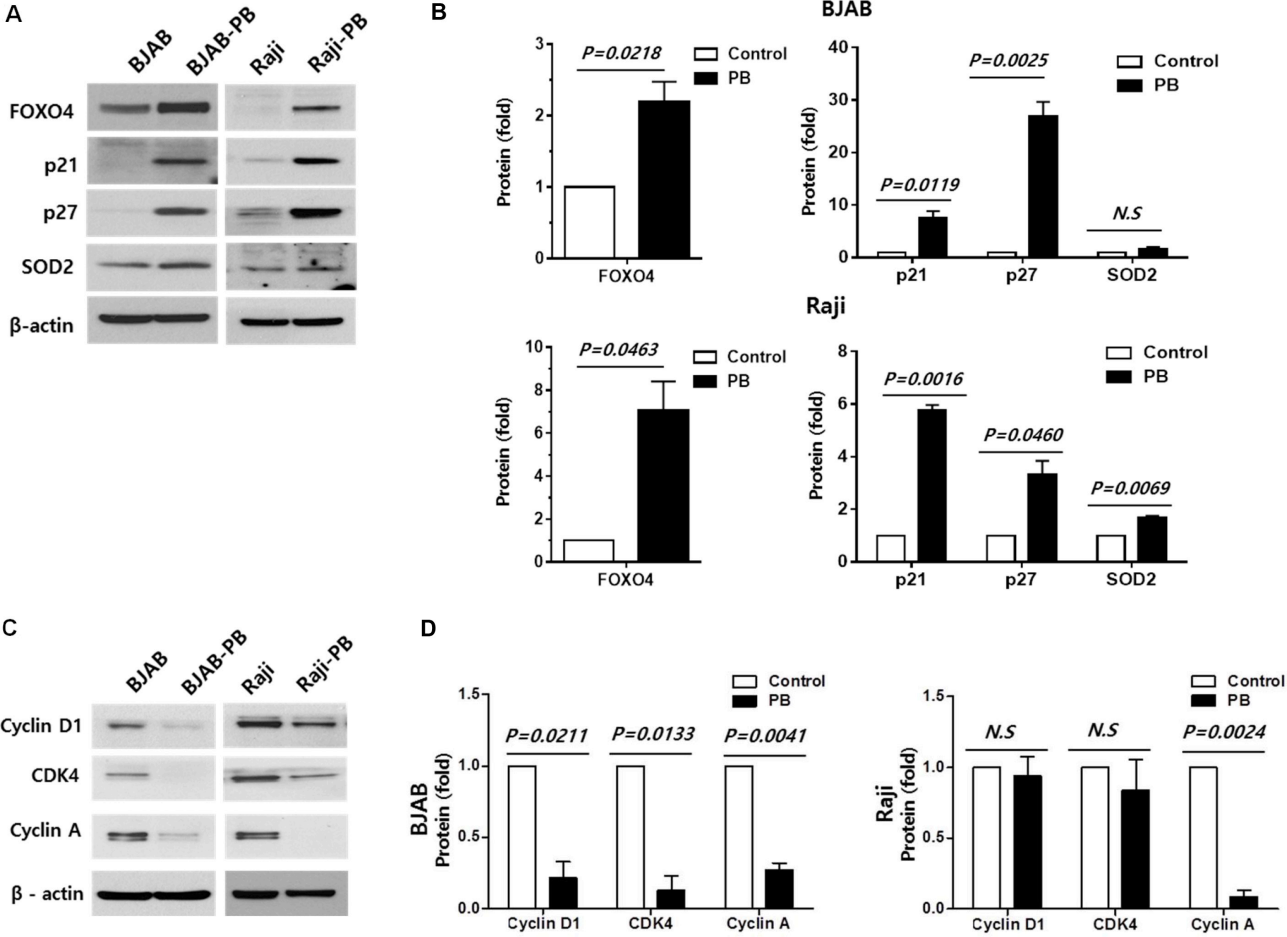
Effect of FOXO4 on the expression of target proteins in phenylbutyrate treated-surviving cells (**A, B**) Phenylbutyrate-treated surviving cells (BJAB-PB and Raji-PB) increase the expression of FOXO4 and its transcriptional targets (p21, p27, and SOD) compared to control cells. (**C, D**) BJAB-PB and Raji-PB cells show decreased expression of cyclin D1, CDK4, and cyclin A compared to control cells.

**Figure 5:**
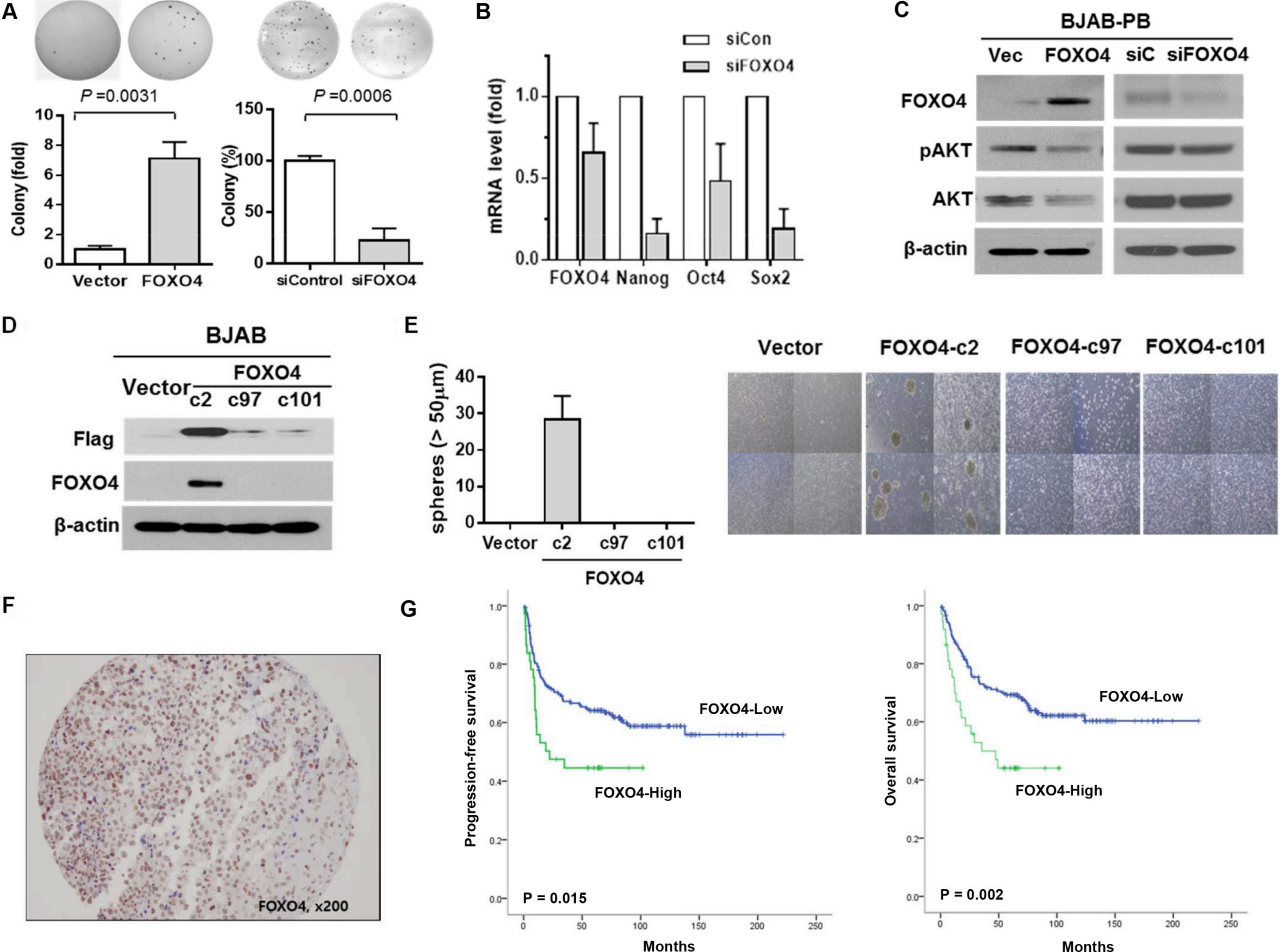
The association of FOXO4 with stem cell properties and prognosis of diffuse large B-cell lymphoma (**A**) Soft agar colony formation in FOXO4-transfected or siFOXO4-transfected BJAB cells shows the amplification of *FOXO4* increase colony forming ability and the knockdown of *FOXO4* decrease colony formation in BJAB cell line. (**B**) Decrease in mRNA levels of Nanog, Oct-4 and Sox-2 in siFOXO4-transfected (siFOXO4) is noted compared to siControl-transfected (siCon) BJAB cells. (**C**) Phosphorylated AKT level is decreased in BJAB-PB cells with FOXO4 overexpression whereas siFOXO4-transfected BJAB-PB cells show the reverse of phosphorylated AKT. (**D**) The western blot shows the expression of FOXO4 protein in a BJAB clone (c2) overexpressing FOXO4. (**E**) Tumor sphere formation is observed from a BJAB clone (c2) overexpressing FOXO4. (**F**) Immunohistochemical staining for FOXO4 in tumor tissue of DLBCL (× 200). (**G**) Kaplan-Meier curves shows superior progression-free survival and overall survival of FOXO4-high group with diffuse large B cell lymphoma than FOXO4-low group. The *P* value is calculated using the log-rank test.

The expression of FOXO4 target proteins (p21, p27, and SOD2) was increased in BJAB-PB and Raji-PB cells compared with control cells (Figure 4A, 4B). On the other hand, the expression of cell-cycle regulatory proteins (cyclin D1, CDK4, and cyclin A) was reduced in BJAB-PB and Raji-PB cells where FOXO4 expression was increased (Figure 4C, 4D). These results represent FOXO4 could up-regulate the expression of p21, p27, and SOD2 whereas FOXO4 down-regulated the expression of cyclin D1, CDK4, and cyclin A in consistent with a previous report [23]. To ascertain whether FOXO4 affects CSC properties of B cell lymphoma, the amplification of *FOXO4* expression increased colony forming ability whereas the knockdown of *FOXO4* decreased colony formation in BJAB cell line (Figure 5A). Likewise, BJAB cell with the knockdown of *FOXO4* showed a significant decrease of stem cell-associated genes, *OCT4, NANOG*, and *SOCS2* (Figure 5B). Phosphorylated AKT level was decreased in BJAB-PB cells with *FOXO4* overexpression whereas siFOXO4-transfected BJAB-PB cells showed the reverse of phosphorylated AKT representing the association of downregulated *FOXO4* expression with the phosphorylation of AKT (Figure 5C). The sphere formation was not detectable in parental BJAB cells or clones with low *FOXO4* expression, however, the sphere formation was markedly increased in the BJAB clone overexpressing *FOXO4* (Figure 5D, 5E).

### Prognostic value of FOXO4 protein expression in DLBCL patients

In total, 211 patients with DLBCL subjected to at least one cycle of chemotherapy with curative intent were analyzed to evaluate the prognostic value of FOXO4 protein expression. The median age of patients was 59 years (19–86 years). The characteristics of patients at diagnosis are summarized in Table 1. FOXO4-high group was defined as DLBCL cases with more than 70% of positively stained nuclei of tumor cells (Figure 5F). Although the optimal cutoff is not established for nuclear staining of FOXO4, we applied the strict definition for FOXO4-high group because some cases displaying weak nuclear positivity for FOXO4 or cytoplasmic staining in molarity of cells might increase the false positive rate. Thus, all cases other than FOXO4-high group were categorized into FOXO4-low group. Overall, 37 patients (18%, 37/211) were classified as FOXO4-high group. FOXO4-high group was significantly associated with unfavorable characteristics, including advanced stage and increased extranodal involvement (*P* < 0.05, Table 1). Thus, the high-intermediate/high-risk group of patients according to the International Prognostic Index (IPI) showed more strong association with FOXO4-high group (32%, 15/47) than the low/low-intermediate risk group (13%, 22/164, *P* = 0.008). Accordingly, the OS and PFS of patients were significantly different according to positivity for FOXO4 (Figure 5G, P < 0.05).

**Table 1:** Characteristics of patients

Characteristics		*n*	(%)	FOXO4		
				Low	High	*P* value
Age (years)	≤ 60	113	(54)	97	16	
	> 60	98	(46)	77	21	0.204
Sex	Male	126	(60)	110	16	
	Female	85	(40)	64	21	0.028
Performance status	ECOG 0/1	190	(90)	161	29	
	ECOG ≥2	21	(10)	13	8	0.015
Ann Arbor stage	I/II	140	(66)	121	19	
	III/IV	71	(34)	53	18	0.037
Serum LDH	Normal	134	(64)	118	16	
	Increased	77	(36)	56	21	0.008
B symptoms	Absence	172	(82)	140	32	
	Presence	39	(18)	34	5	0.489
Bone marrow involvement	Absence	197	(93)	164	33	
	Presence	14	(7)	10	4	0.276
Extranodal involvement	0/1	165	(78)	142	23	
	≥ 2	46	(22)	32	14	0.015
IPI	L / LI	164	(78)	142	22	
	HI / high	47	(22)	32	15	0.008

## DISCUSSION

Our study used an *in vitro* model mimicking primary refractory lymphoma cells by isolating the cell fraction that survived after high-dose drug treatment. This subset of lymphoma cells survived after treatment with high-dose doxorubicin and phenylbutyrate exhibited stem cell-like properties compared to the untreated parental cell population including the expression of stem cell markers, colony and sphere forming activity and resistance to chemotherapeutic agents. Because phenylbutyrate was reported to induce pluripotency-associated genes [15], this small population of lymphoma cells might be enriched with cells having stem cell-like properties (so-called CSCs). The presence of CSCs in tumor cells have been demonstrated by the application of ALDH assay and side-population analysis based on characteristics of normal stem cells such as high expression of adenosine triphosphate-binding cassette transporters protecting against cytotoxic agents [19–21]. Our study also showed higher proportion of ALDH-positive cells and side-population in this subset of cells survived after the treatment with IC_90_ dose of phenylbutyrate than control cells (Figure 2E, 2F). Taken together, these findings were consistent with that the small subset of cells could have stem cell-like properties leading to treatment failure in lymphomas.

Given this subset of cells having stem cell-like properties was resistant to chemotherapeutic agents in this study, the comparison of gene expression profiles between treatment-surviving and untreated control cells was performed to find a marker representing the cell populations with drug resistance. Our comparison revealed the increased expression of 41 genes in the four different lymphoma cell lines (BJAB, Raji, Daudi, Toledo, Figure 3A). These gene were commonly overexpressed in doxorubicin- and phenylbutyrate-treated surviving cells. Among them, we chose *FOXO4* as a candidate gene for indicating the cell fraction with stem cell-like properties because previous studies have suggested the role of FOXO transcription factors in the maintenance of stem cell characteristics [16–18]. The increased expression of *FOXO4* was also demonstrated in other DLBCL cell lines (OCI-Ly10, Riva, and U2932) when doxorubicin- and phenylbutyrate-treated surviving cells were analyzed (Supplementary Figure S3). Furthermore, primary cells from refractory DLBCL patients showed increased expression of *FOXO4* compared to that of patient with complete response (Figure 3E). These results might support the association of *FOXO4* expressing cells with CSCs leading to drug resistance. Thus, overexpression of FOXO4 might have a potential role as a predictor of treatment failure in DLBCL.

The FOXO family transcription factors consisting of four members, FOXO1, FOXO3, FOXO4, and FOXO6, are reported to have various roles in the regulation of stress response and oncogenesis [24]. Previous studies reported that FOXOs protect cells under stress conditions (e.g., oxidative stress, serum deprivation or hypoxia) and contribute to cancer progression. Thus, FOXOs were reported to promote invasion and metastasis of colon and breast cancers [25–27]. FOXOs also could contribute to the maintenance of leukemia-initiating cells in chronic myeloid leukemia [17, 18, 28]. Likewise, the depletion of FOXOs could induce leukemic cell maturation and cell death in acute myeloid leukemia [16]. In our experiments, expression levels of FOXO4 and its transcriptional targets (p21, p27 and SOD2) were low or undetectable in untreated control cells but highly upregulated in treatment-surviving cells. Additionally, FOXO4 expression led to enhanced transcription of stem cell factors and self-renewal activity of lymphoma cells whereas its knockdown had opposite effects. The major mechanism of FOXO regulation is associated with phosphorylation by Akt and other kinases [29]. Phosphorylation of FOXO factors lead to their nuclear export and abrogation of transcriptional activity. Accordingly, we analyzed the association of FOXO4-positive cells with treatment failure using an archived tumor tissue array of DLBCL patients. In our study, the survival outcome of FOXO4-high group was significantly worse than that of FOXO4-low group (Figure 5G).

In conclusion, a small subset of lymphoma cells surviving treatment with doxorubicin or phenylbutyrate showed stem cell-like properties including the expression of stem cell markers, increased colony and sphere formation and resistance to chemotherapeutic agents. The overexpression of FOXO4 was found in these surviving cells, and DLBCL patients with FOXO4-positive tumor cells had poor prognosis. Thus, our results might suggest a role of FOXO4 as an indicator for the presence of cell population enriched with CSCs leading to drug resistance and treatment failure.

## MATERIALS AND METHODS

### Cell lines and cultures

The following human B-cell lymphoma cell lines were used for culture: three Burkitt's lymphoma cell lines (BJAB, Raji, Daudi), one germinal center type DLBCL line (Toledo), and three activated B-cell type DLBCL cell lines (Riva, U2932, and OCI-Ly10). Toledo, Daudi and Raji were purchased from ATCC (Rockville, MD), and Riva and U2932 from DSMZ (Braunschweig, Germany). BJAB cells were obtained from Dr. W.S. Kim (Sungkyunkwan University) and OCI-Ly10 from Dr. Y.K. Jeon (Seoul National University Hospital). Patient-derived primary cells were acquired from malignant pleural effusion or ascites of patients with DLBCL. Seven patients had refractory disease whereas one patient achieved complete response to the 1^st^ line chemotherapy. Cells were cultured in RPMI 1640 supplemented with heat-inactivated 10% fetal bovine serum, penicillin and streptomycin (Gibco BRL, Grand Island, NY), except OCI-Ly10, which was cultured in Iscove's modified Dulbecco's medium.

### Flow cytometry

Cells were suspended at a concentration of 5 × 10^5^ cells in Dulbecco's Phosphate Buffered Saline (DPBS) containing 2% fetal bovine serum (FBS) and incubated with CD45 and CD19 or isotype control (BD Bioscience) for 1 h at 4°C. After washing with PBS, cells were analyzed via flow cytometry (FACSverse, BD Biosciences, San Jose, CA, USA).

### May-Grünwald-Giemsa staining

Cells (2 × 10^4^ cells) were fixed in methanol for 2–3 min and stained with May-Grünwald solution for 15 min, followed by Giemsa solution for 10 min. After washing with H_2_O, cell morphology was examined under a microscope.

### Gene expression analysis following drug treatment

The IC_90_ concentration of doxorubicin and phenylbutyrate (Enzo life science, INC, Farmingdale, NY, USA) was determined as the dose that induced 90% tumor cell death. For drug treatment, four cell lines (BJAB, Raji, Daudi and Toledo) were seeded at a density of 5 × 10^6^ cells per 150 mm dish and incubated with 300 nM doxorubicin or 8 mM phenylbutyrate for 48 h. For the negative control, the same cell lines were incubated with DPBS for 48 h. Cell viability was determined by cell counting using a hemocytometer (Hausser Scientific, Horsham, PA, USA) after staining with 0.4% Trypan blue solution (Sigma-Aldrich, Inc., St Louis, MO, USA). Viable cells were sorted using a BD FACSAria™ III cell sorter, and total RNA extracted with TRIzol (Invitrogen Life Technologies, Carlsbad, CA) and amplified using the TargetAmp-Nano Labeling Kit for Illumina Expression BeadChip (EPICENTRE, Madison). Labeled RNA samples were prepared according to the manufacturer's instructions (Illumina, Inc., San Diego), and array signals detected with Amersham fluorolink streptavidin-Cy3 (GE Healthcare Biosciences, Little Chalfont, UK). Raw data were extracted using the software provided by the manufacturer (Illumina GenomeStudio v2011. 1 (Gene Expression Module v1.9.0)). Array probes with detection *p-*values ≥ 0.05 (similar to signal-to-noise) in over 50% samples were filtered out. We applied a filtering criterion for data analysis. Thus, a higher signal value was required to obtain a detection *p-*value < 0.05. The selected gene signal value was transformed by the logarithm and normalized with the quantile method. The statistical significance of expression data was determined based on fold change. Gene Enrichment and Functional Annotation analyses were performed with DAVID (http://david.abcc.ncifcrf.gov/). All data analyses and visualization of differentially expressed genes were conducted using R 2.15.1 (www.r-project.org).

### Cell survival and colony formation assays

Cells were seeded at a density of 5 × 10^4^ per well in 24-well plates and incubated with 0–1 μM doxorubicin, 0–3 μM prednisolone for 24 h and 0–100 ug/ml rituximab for 72 h. After this period, cells were stained with 0.4% trypan blue solution (Sigma-Aldrich, Inc) and counted using a hemocytometer (Hausser Scientific, Horsham, PA). Data were expressed as percentage cell proliferation, taking the number of living cells incubated with DPBS as 100% reference. For colony formation, cells were seeded in triplicate at a density of 5 × 10^3^ cells per well in six-well plates. The base agar was 0.3% agar containing RPMI 1640 with 10% FBS while the top agar was 0.6% agar in the same culture medium. A 2 ml aliquot of medium was added, and the plates incubated at 37°C. Colonies were counted after 2 weeks.

### Side population analysis

The cells were suspended at 1 × 10^6^ cells of RPMI 1640 medium containing 2% FBS and 10 mM HEPES buffer and incubated with Hoechst 33342 dye (Sigma-Aldrich, Inc) at a final concentration of 5 μg/mL at 37°C for 60 minutes in the dark, either alone or in the presence of 100 μM verapamil (Sigma-Aldrich). After washing with PBS, cells were analyzed by flow cytometry (FACSAria SORP, BD biosciences). Hoechst 33342 dye was excited with a UV laser at 355 nm, and fluorescence emission from Hoechst blue was collected with a 450/50 band pass (BP) filter and Hoechst red was collected with a 670 LP filter.

### Aldehyde dehydrogenase (ALDH) assay

ALDH activity was measured using the ALDEFLUOR kit (Stemcell Technologies, Vancouver, BC, Canada) according to the manufacturer's protocol. The cells (1.0 × 10^6^ cells) were incubated with ALDEFLUOR assay buffer for 60 min at 37°C, either alone or in the presence of the specific ALDH1 enzyme inhibitor diethylaminobenzaldehyde (DEAB), which was a negative control. After washing with PBS, cells were analyzed by flow cytometry (FACSverse, BD Biosciences, San Jose, CA, USA).

### Reverse transcriptase (RT)–PCR

Total RNA was extracted using TRIzol (Invitrogen Life Technologies, Carlsbad, CA, USA) according to the manufacturer's protocol. For reverse transcription, 2 μg RNA was converted to cDNA using an Omniscript RT kit (Qiagen, Valencia, CA, USA). The generated cDNA was amplified using specific primers, with glyceraldehyde 3-phosphate dehydrogenase (*GAPDH*) as an internal normalization control. Each PCR cycle consisted of denaturation at 94°C for 30 sec, primer annealing at 58°C for 30 sec, and extension at 72°C for 30 sec. Sample was amplified for 35 cycles, and an additional extension step carried out at 72°C for 5 min. The specific primer sequences were as follows: FOXO4 forward, 5′- TGG GCT CAA TCT CAC CTC TTC C -3′ and FOXO4 reverse, 5′- AGA AGC ACC CTT CTC CTG CTG A -3′; NANOG forward, 5′-AAA GAA TCT TCA CCT ATG CC-3′ and NANOG reverse, 5′-GAA GGA AGA GGA GAG ACA GT-3′; OCT4 forward, 5′-CGA CCA TCT GCC GCT TTG AG-3′ and OCT4 reverse, 5′-CCC CCT GTC CCC CAT TCC TA-3′; SOX2 forward, 5′- CCT CCG GGA CAT GAT CAG -3′ and SOX2 reverse, 5′- TTC TCC CCC CTC CAG TTC -3′; GAPDH forward, 5′-ACA GTC AGC CGC ATC TTC TT -3′ AND GAPDH REVERSE, 5′- GAC AAG CTT CCC GTT CTC AG -3′.

### Quantitative real-time PCR

Real-time quantitative polymerase chain reaction (qPCR) primers for FOXO4, Nanog, OCT4, SOX2 and β-actin were purchased from Applied Biosystems (TaqMan Gene Expression Assays, Austin, TX), and reactions performed according to the manufacturer's protocol. Reactions were carried out in an ABI7900HT system, and the results expressed as fold change calculated with the ΔΔCt method relative to the control sample. β-Actin was used as the internal normalization control.

### Plasmids, siRNAs and transfection

FOXO4-Flag overexpression constructs were generated as follows: FOXO4 cDNA was obtained from Addgene (plasmid 17549) and subcloned into a pCMV tag4C vector to generate pCMV tag4C FOXO4-Flag. BJAB was transfected with pCMV tag4C FOXO4-Flag or empty vector using the Amaxa Nucleofector™ kit T and the corresponding Amaxa Nucleofector™ System (Amaxa, Gaithersburg, MD). Stable cell lines overexpressing FOXO4 were selected with changes of fresh medium containing G418 (500 μg/ml) for 4 weeks. FOXO4-targeting and control siRNA were purchased from Dharmacon (Thermo Scientific, Waltham, MA).

### Western blot

Whole cell lysates were prepared using ice-cold RIPA buffer (0.5% sodium deoxycholate, 1% Nonidet P-40, 150 mM NaCl, 50 mM Tris [pH 7.5], 0.1% sodium dodecyl sulfate [SDS] and 1 mM phenylmethylsulfonyl fluoride [PMSF]) and cleared by microcentrifugation (14 000 rpm for 30 min at 4°C). The protein concentration in each sample was estimated with the BCA Assay. In total, 30–40 μg protein sample was electrophoresed on a 12% SDS polyacrylamide gel (SDS-PAGE) and electroblotted onto nitrocellulose membranes. After 1 h of incubation in blocking solution (5% non-fat milk), membranes were exposed overnight at 4°C to primary antibodies, included those against FOXO4, pAKT, AKT (Cell Signaling, Danvers, MA), p27 (BD Pharmingen, San Diego, CA), p21, cyclin D1, cyclin A, CDK4 and β-actin (Santa Cruz Biotechnology, Santa. Cruz, CA, USA). The blot was washed with TBST buffer (50 mM Tris [pH 7.5], 150 mM NaCl. 0.05% Tween 20) and further exposed to horseradish peroxidase-conjugated secondary antibody for 1 h at room temperature. Proteins were visualized using enhanced chemiluminescence (ECL) reagent (Amersham Pharmacia Biotech, Arlington Heights, IL).

### Immunohistochemistry

FOXO4 expression was analyzed in archived tissue arrays of DLBCL patients from Samsung Medical Center pathologically diagnosed with DLBCL and treated using systemic chemotherapy with curative content. Clinical data, including disease and survival status, were updated in December 2015, and written informed consent from patients exempted by the Samsung Medical Center Institutional Review Board due to the retrospective nature of the study. Immunohistochemical analysis of FOXO4 was performed on formalin-fixed, paraffin-embedded, 4 μm thick tissue sections. Tissue sections were deparaffinized three times in xylene for a total of 15 min, and antigen retrieval performed for 20 min with ER1 buffer (pH 6.0) in 97°C. After endogenous peroxidase blocking for 5 min, tissues were incubated with primary polyclonal antibody against FOXO4 (1:1000; GTX50500; GeneTex, Irvine, CA, USA) for 15 min using a BOND-MAX autoimmunostainer (Leica Biosystems, Melbourne, Australia) for 15 min. The secondary antibody reaction was performed using a BOND-MAX autoimmunostainer (Leica Biosystems, Melbourne, Australia) with the BOND Polymer Refine Detection system (DS9800; Vision BioSystems, Melbourne, Australia) for 10 min. Positivity for FOXO4 was determined by comparing with the normal positive control from the tonsillar germinal center. In every staining set, a negative control was included in which primary antibodies and probes were omitted. Stained slides were reviewed, and the extent of tissue expression determined by pathologists (M.H. and Y.H.K).

### Statistical analysis

Categorical variables were analyzed with Fisher's exact test. Progression-free survival (PFS) was calculated from the date of diagnosis to the first day of progression, relapse or death from any cause. Overall survival (OS) was calculated from the date of diagnosis to death. Survival curves were estimated with the Kaplan–Meier method and compared using the log-rank test. *P* values < 0.05 were considered significant, and two-sided tests used in all calculations. Statistical analyses were performed using the IBM PASW version 18.0 package (SPSS Inc., Chicago, IL, USA).

## SUPPLEMENTARY MATERIALS FIGURES AND TABLES


