# Supplementary material for: FOXO4 expression is related to stem cell-like properties and resistance to treatment in diffuse large B-cell lymphoma
Source: Oncotarget. 2016 Nov 29;8(2):2466–76. doi: 10.18632/oncotarget.13690 (PMC5356816; doi:10.18632/oncotarget.13690)
Supplement: Supplementary file 1 [file oncotarget-08-2466-s001.pdf]

# Forkhead box O4 expression is related to stem cell-like properties and resistance to treatment in diffuse large B-cell lymphoma

## Supplementary Materials

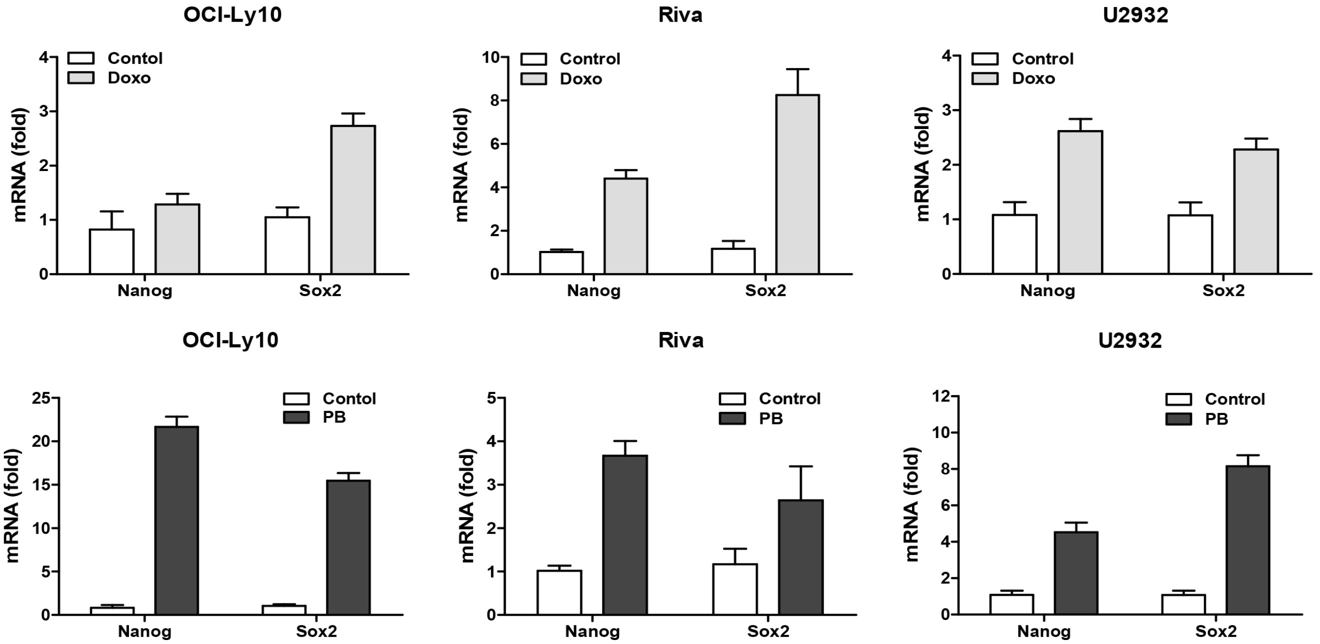


**Supplementary Figure S1: The mRNA expression of Nanog and SOX2 is increased in surviving cells after IC90 treatment with doxorubicin and phenylbutyrate compared to control cells in other B-cell lymphoma cell lines (OCI-Ly10, Riva, U2932).**

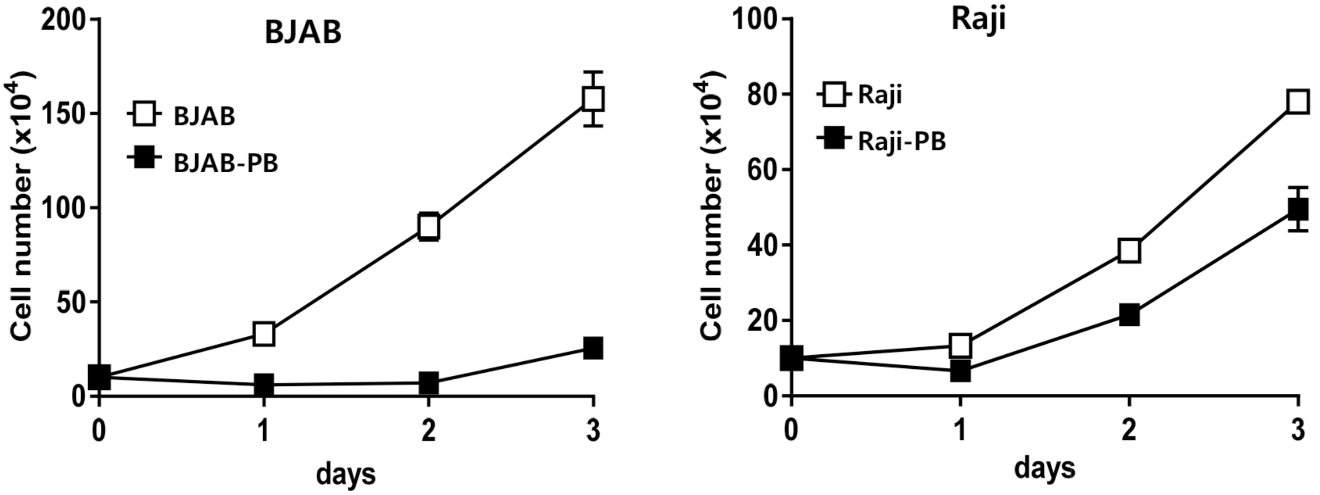


**Supplementary Figure S2: Cell growth kinetics of non-treated and treatment-surviving cells.** The values represent means ± SD of three independent experiments. After treatment with phenylbutyrate (8 mM) for 48 h, surviving live cells are sorted via flow cytometry and cultured. Cell growth is monitored every 24 h for 3 days. The cell number is assessed based on trypan blue staining. Data represent means ± SD.

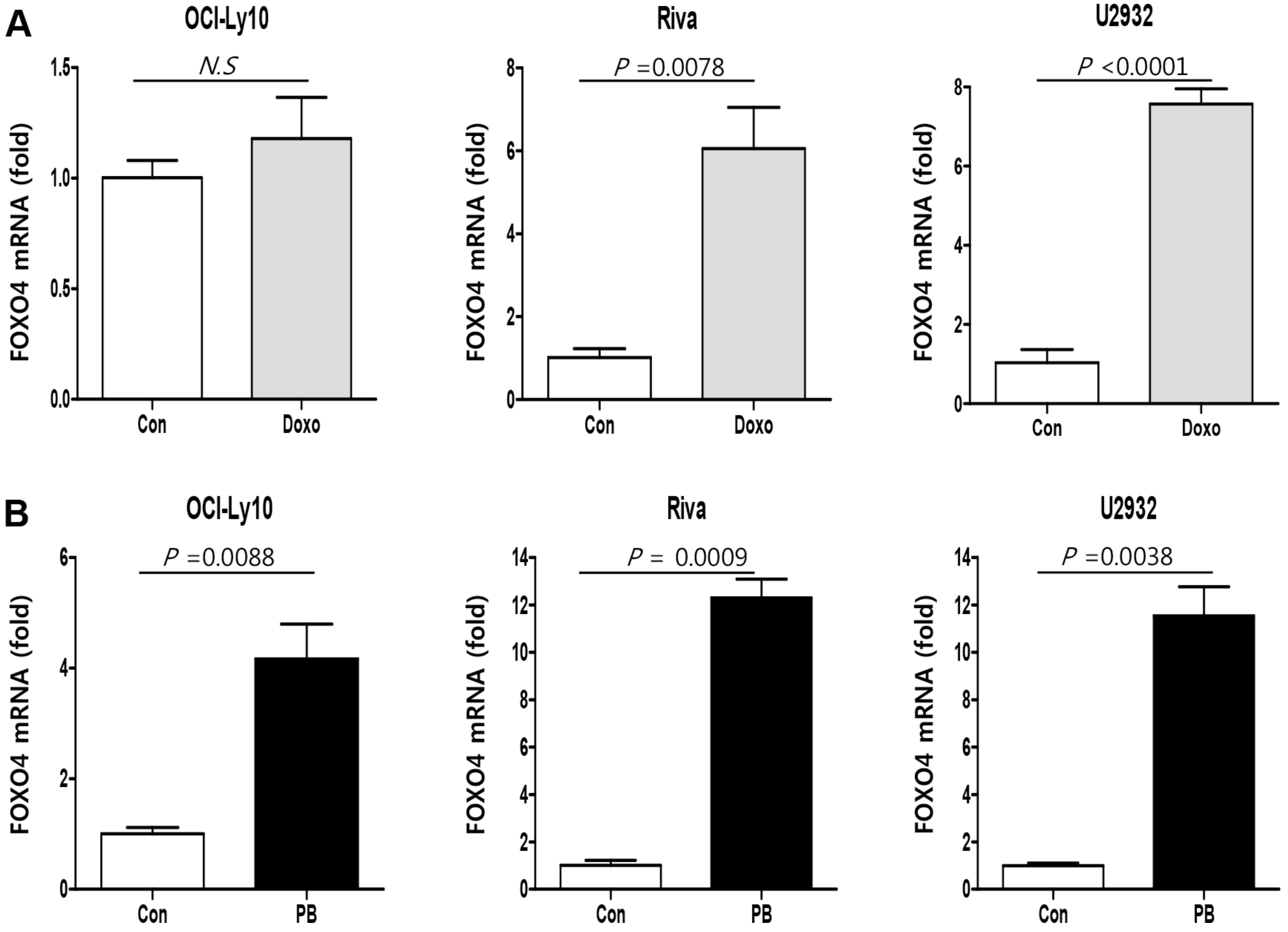


**Supplementary Figure S3: FOXO4 mRNA levels are upregulated in treatment-surviving B cell lymphoma cells.** (**A**) OCI-Ly10 cells are treated with doxorubicin (300 nM) for 48 h, and Riva and U2932 cells treated with Doxorubicin (3 uM) for 72 h. (**B**) Phenylbutyrate (8 mM) treatment is performed for 24 h. Non-treated cells (control) and respective treatment-surviving cells are collected and FOXO4 mRNA expression evaluated using RT-qPCR. Data represent means ± SEM of three independent experiments.

**Supplementary Table S1: Proportion of viable cells after treatment with doxorubicin or phenylbutyrate**

| Cell line | Doxorubicin | Phenylbutyrate |
|---|---|---|
| **BJAB** | 9.48 ± 2.37% | 10.28 ± 1.87% |
| **Raji** | 7.45 ± 3.77% | 6.54 ± 2.22% |
| **Daudi** | 6.15 ± 0.93% | 15.57 ± 0.93% |
| **Toledo** | 9.61 ± 5.12% | 6.32 ± 1.47% |
| **OCI-Ly10** | 10.75 ± 1.95% | 8.6 ± 3.35% |
| **Riva** | 10.49 ± 0.17% | 6.13 ± 1.64% |
| **U2932** | 10.12 ± 3.00% | 6.43 ± 1.48% |

**Supplementary Table S2: Commonly upregulated genes in treatment-surviving cells**

| Gene Symbol | Gene Title | Gene Symbol | Gene Title |
|---|---|---|---|
| *ACTA2* | smooth muscle aortic alpha-actin | *HLA-H* | MHC complex, class I, H |
| *APOBEC3F* | Apolipoprotein B mRNA editing enzyme | *ID2* | Inhibitor of DNA binding 2 |
| *C9orf9* | Chromosome 9 open reading frame 95 | *IFI6* | Interferon alpha-inducible protein 6 |
| *CCR7* | Chemokine receptor 7 | *JUN* | Jun proto-oncogene |
| *CDKN1A* | Cyclin-dependent kinase inhibitor 1A | *KLF6* | Kruppel-like factor 6 |
| *COL9A2* | Collagen, type IX, alpha 2 | *LOC727820* | Hypothetical protein |
| *DUSP1* | Dual-specificity phosphatase 1 | *MX1* | Myxovirus resistance 1 |
| *EPSTI1* | Epithelial-stromal interaction1 | *MXD4* | MAX dimerization protein 4 |
| ***FOXO4*** | Forkhead box O4 | *N4BP2L1* | EDD4 binding protein 2-like 1 |
| *GNS* | Glucosamine N-acetyl-6-sulfatase | *NEU1* | Neuraminidase 1 |
| *HCP5* | HLA complex P5 | *NPC2* | Niemann-Pick disease, type C2 |
| *HIST1H1C* | Histone cluster 1,H1c | *OAS2* | Oligoadenylate synthetase 2 |
| *HIST1H2A* | Histone cluster 1, H2ac | *OPTN* | Optineurin |
| *HIST1H2BD* | Histone cluster 1, H2b | *PRIC285* | PPARa interacting complex 285 |
| *HIST1H2BK* | Histone cluster 1, H2bk | *PSAP* | Prosaposin |
| *HIST1H4H* | Histone cluster 1, H4h | *STAT2* | STAT2 |
| *HIST2H2AA3* | Histone cluster 2, H2aa3 | *TCTN1* | Tectonic family member 1 |
| *HIST2H2BE* | Histone cluster 2, H2be | *TP53INP1* | TP53 inducible nuclear protein 1 |
| *HLA-A* | MHC class I, A | *TSGA10* | Testis specific, 10 |
| *HLA-B* | MHC class I, B | *YPEL5* | Yippee-like 5 |
| *HLA-F* | Major histocompatibility complex, class I, F | | |
